# Vitamin D Levels Are Associated with Expression of SLE, but Not Flare Frequency

**DOI:** 10.1155/2014/362834

**Published:** 2014-11-24

**Authors:** Marline L. Squance, Glenn E. M. Reeves, Huy A. Tran

**Affiliations:** ^1^Faculty of Health and Medicine, University of Newcastle, Callaghan, NSW 2308, Australia; ^2^Faculty of Science and Information Technology, University of Newcastle, Callaghan, NSW 2308, Australia; ^3^Autoimmune Resource and Research Centre, 2nd Floor HAPS Building, John Hunter Hospital, New Lambton, NSW 2305, Australia; ^4^Pathology North, Hunter New England, New Lambton, NSW 2305, Australia; ^5^Hunter Medical Research Institute, New Lambton, NSW 2305, Australia

## Abstract

This study explores links between vitamin D deficiency (25(OH)D = 50 nmol/L) and serological autoimmunity (ANA > 1 : 80) and frequency of self-reported flares (SRF) in participants with clinical autoimmunity (SLE). 25(OH)D levels of 121 females were quantified and compared. The cohort consisted of 80 ACR defined SLE patients and 41 age and sex matched controls. Association analysis of log2 (25(OH)D) levels and ANA 80 positivity was undertaken via two-sample *t*-tests and regression models. Significant differences were found for 25(OH)D levels (mean: control 74 nmol/L (29.5 ng/ml); SLE 58 nmol/L (23.1 ng/ml), *P* = 0.04), 25(OH)D deficiency (*P* = 0.02). Regression models indicate that, for a twofold rise in 25(OH)D level, the odds ratio (OR) for ANA-positivity drops to 36% of the baseline OR. No link was found between SRF-days and 25(OH)D levels. Our results support links between vitamin D deficiency and expression of serological autoimmunity and clinical autoimmunity (SLE). However, no demonstrable association between 25(OH)D and SRF was confirmed, suggesting independent influences of other flare-inducing factors. Results indicate that SLE patients have high risk of 25(OH)D deficiency and therefore supplementation with regular monitoring should be considered as part of patient management.

## 1. Background

Systemic lupus erythematosus (SLE) is a systemic autoimmune illness with a complex and multifactorial pathogenesis [1]. Patients can exhibit a wide range of symptoms including increased photosensitivity to ultraviolet radiation (UV) exposure, combined with immunological markers of antinuclear antibody positivity. It is thought that UV exposure may be a catalyst to symptom exacerbation or flare events [2–4] and therefore SLE patients are often advised to adopt sun-protective measures of using both physical and chemical barriers on a routine basis.

Although the use of UV protective measures is important as a management strategy, it is undertaken with reservation, as adopting such measures may result in vitamin D deficiency and insufficiency reducing individual patient capacity to maintain vitamin D synthesis within the skin. Mechanisms of UV-related influences in SLE range from induction of anti-double stranded DNA (anti-dsDNA) autoantibody production resulting in skin lesion exacerbation [3] through the possibility that vitamin D effects may be inhibited by SLE patient serum autoantibodies (anti-vitamin D) [5]. It is clear that vitamin D is an important hormone with immunomodulating properties [6–8] and has a vital role in a large number of biologic and biochemical pathways [3, 9, 10].

**Figure 1:**
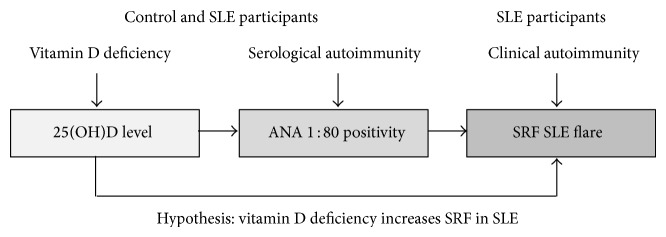
Proposed pathogenic pathway.

Deficiency of vitamin D is also demonstrated in association with a range of immune disturbances, including allergy and autoimmune illnesses such as SLE, ulcerative colitis, rheumatoid arthritis, and multiple sclerosis [9, 11–15]. This study further explores possible links between vitamin D deficiency and autoimmunity as expressed by two parameters—antinuclear antibody positivity (ANA, across normal controls and SLE patients) and frequency of self-reported flares (SRF) in participants with confirmed autoimmune illness (SLE). Our study incorporated a group of matched controls, so that important factors modulating the risk of autoimmunity could be assessed. The proposed study's pathogenic pathway is presented within Figure 1.

In our cohort of patients with SLE, we set out to explore the determinants of flare events from a patient's perspective with the primary aim being to determine whether lower vitamin D levels were predictive of an increased risk of SRF in SLE patients. The secondary goal was to assess whether any such link was mediated by the serological expression of autoimmunity, as reflected by ANA positivity with a cutoff of ANA 1 : 80.

## 2. Patients and Methods

The overall hypothesis of this study was that, through immunomodulating activity, vitamin D (25(OH)D) levels could influence the risk of an SLE patient experiencing and reporting a flare event. The hypothesis proposes a 2-step pathway: (i) from low vitamin D levels to expression of serological autoimmunity (expressed by ANA-positive ratio ≥1 : 80) and (ii) from ANA positivity to frequent flare events. The overall pathogenic pathway (Figure 1) examined associations between levels of 25(OH)D and SRF-days.

Vitamin D (25(OH)D) levels of a cohort of 121 female participants were quantified and compared with reference to vitamin D deficiency, insufficiency, or normal levels [3] across the cohort. Methods specific to 25(OH)D quantification and analysis are outlined below. The cohort consisted of 80 SLE patients as defined by the American College of Rheumatology (ACR) classification criteria for SLE [16] and 41 age and sex matched control study participants. Participants were Australian and of Caucasian ethnicity: they completed study specific questionnaires in regard to relevant demographics and daily living practices including hours spent in the outdoors environment and use of sun protective products of sunscreen (SS) and foundation makeup (FM). Vitamin D supplementation for all participants and use of other immunotherapy medications (ITM) which have photosensitizing properties such as methotrexate, hydroxychloroquine, prednisolone, and azathioprine were also recorded for SLE participants. ITM was considered as a combined group and also as individual interactive covariates. Supplementation of vitamin D was also considered as a separate covariate due to its reported immunomodulation properties [6, 17].

All participants had blood collected for assessment of general measures of health and immunological markers relevant to autoimmune illness and specifically SLE diagnosis, including ANA, DNA, and ENA testing. ANA positivity was recorded with ratios ≥1 : 80 and used to investigate any link between vitamin D levels and serological autoimmunity.

SLE participants also shared their flare experience in an interview designed to examine lupus flare history over the 12-month period prior to interview. Flare data was of a self-reported retrospective nature with SRF specific methods detailed previously [18] and briefly summarised for this paper. SRF data, in particular, calculated flare days and SRF status was analysed to explore any associations with ANA positivity. Finally, possible links between vitamin D levels and likelihood of SLE flares were explored.

Ethical review and approval of study according to Declaration of Helsinki 2008 [19] were received from the University of Newcastle and Hunter New England Health Human Research Ethics Committees.

### 2.1. Study Population

Participants were recruited from the Hunter and Central Coast areas of New South Wales (NSW), Australia. SLE participants were recruited through the Autoimmune Resource and Research Centre (ARRC) and private immunology clinics. Inclusion criteria were specified as being 18–80 years of age and having a confirmed diagnosis of SLE. Confirmation of participant SLE diagnosis was obtained through auditing of health records according to ACR SLE classification [16]. Limited numbers of males and participants from ethnic diverse backgrounds responded to recruitment invitations, so the final study cohort reflects a female largely homogeneous Caucasian population. Control participants were recruited through the volunteer research participant database of the Hunter Medical Research Institute (HMRI). Recruitment inclusion criteria for control participants were specified as being 18–80 years of age, female, and not having an autoimmune illness.

### 2.2. Data Collection

As part of study specific questionnaires of a wider study (environmental determinants of lupus flares (EDOLF)), participants self-reported their personal medical history including medication and supplement use, home environment characteristics, and lifestyle practices. Of particular interest were usage patterns of ultraviolet (UV) protective products such as SS and FM, as well as hours spent undertaking activities in the outdoors during daylight hours.

Participants attended a scheduled study clinic appointment in the 2006 spring season (September–November) to collect questionnaires and complete study requirements including diagnostic pathology testing and assessment of relevant health measurements. Biological samples were collected by a qualified phlebotomist, processed immediately, and stored at −20°C until specific analysis was required. Height and weight measures were used for calculation of body mass index [20].

SLE participants were also requested to participate in an extended structured interview to explore their personal experience and perception of their flare events. Flares were not assessed within a full physician directed clinical assessment; therefore more traditional SLE activity assessment tools (e.g., SLEDAI) could not be used. Study flare assessment involved a medical researcher following a study script inclusive of a novel flare definition [21] and a series of questions prompting recount of the number and duration of SRF, symptoms, and management over the previous 12 months. Comprehensive methods for flare assessment used in this study have been detailed elsewhere [18]. A calculation of flare days was made for the study period by multiplying the SRF number and average event length.

### 2.3. Foundation Makeup (FM) and Sunscreen (SS) Use

Due to the UV protective properties of FM and SS [22] and the potential relationship between use of these products and vitamin D status [23], this study collected specific data regarding participant use of these products. Product use was calculated by combining self-reported use count values within a scale of “daily (365),” “weekly (52),” “monthly (12),” “yearly (1),” and “do not know (0).” Use was reported within a home cleaning and maintenance product list (HCMPL) of the wider EDOLF study and documenting home cleaning and personal care products used over the study year. HCMPL reported products were categorised based on intended purpose of named product, with a combination category of “makeup” (FMSS) including the topical lotions of FM and SS only.

### 2.4. Outdoor Hours

Study year outdoor hours were calculated from participant reported estimates of average number of hours spent on a regular weekday and also weekend day in the outdoors environment.

### 2.5. Vitamin D (25(OH)D) Analysis

Blood samples were collected and centrifuged at 3500 rpm for 15 minutes prior to freezing at −20°C. Serum was thawed prior to analysis for quantification of 25-hydroxyvitamin D (25(OH)D) via a manual radioimmunoassay (Immunodiagnostics Systems Limited, UK) using ^125^iodine-labelled 25-hydroxyvitamin D. The method measures total (D_2_ plus D_3_) 25-hydroxyvitamin D. Serum calcium was quantified with the RXL Dimension or Vista 1500 platforms (Siemens Healthcare Diagnostics, USA). Analysis was performed in a tertiary referral laboratory, fully accredited to the standard required by the National Australian Testing Authority (NATA) (Pathology North, Hunter New England). Vitamin D status in analysis was categorised as deficiency (50 nmol/L (≤20 ng/mL)), insufficiency (52.5–72.5 nmol/L (>20.0 ≤29 ng/mL)), and normal (72.5 nmol/L (>30 ng/mL)) [3].

All samples were collected within spring season of 2006 (September–November), average daily temperature 24.4°C (range 21.5–28.5°C), and average daily global solar exposure 20.0 MJ/m^2^ (range 17.2–23.2 MJ/m^2^) [24]. Longitude and latitude of study area ranged between 151.16E and 152.01E and 32.68S and 33.5S.

### 2.6. ANA Analysis

Antinuclear antibody testing used indirect immunofluorescence on Hep-2 slides (*Immuno Concepts*, Sacramento, USA) according to manufacturer's instructions, with a screening dilution of 1 : 40 and doubling serial dilution to 1 : 2560. Enzyme-linked immunosorbent assays (ELISA) were used to measure antibodies to DNA (*Trinity Biotech*, Co. Wicklow, Ireland) and ENA (*Euroimmun*, Lübeck, Germany), with assays performed on the automated* Trituras* platform (*Diagnostic Grifols*, Barcelona, Spain).

### 2.7. Other Risk Factors

Information on participant demographics, comorbid medical history, and general health and wellbeing were collected along with SLE participant nominated date of diagnosis which was crosschecked within the health record audit. Participant socioeconomic status (SES) as optioned categories of “above Australian average,” “Australian average,” and “below Australian average” was also collected. Current smoking status was captured as a dichotomised “yes” or “no” response and crosschecked with serum cotinine testing.

### 2.8. Statistical Methods

Demographic information of participant groups was summarised with the use of simple descriptive statistics. Associations between participant groups and independent variables were analysed via one-way ANOVA for continuous variables and Fisher's exact test of independence for dichotomous variables. Separate analysis was performed for use of vitamin D supplementation and 25(OH)D level statuses. Shapiro-Wilk testing did not confirm Gaussian distribution; therefore, to allow intuitive interpretation of any association, data was normalised through log2 transformation of (25(OH)D) levels. Association analysis of log levels and ANA 80 positivity was undertaken via two-sample *t*-test with unequal variances, as well as linear regression with dependent variable log2(25(OH)D) levels and logistic regression with ANA positivity as a binary dependent variable.

SLE participant SRF-days and 25(OH)D level relationship was assessed with negative binomial regression models due to overdispersion of flare data. All variables of interest were included in the initial model with a backward stepwise approach with covariates of age; diagnosis years; ACR criteria, outdoors hours; SES; BMI; FMSS, vitamin D supplementation, use of hormones, and ITM. All significant *P* values (≤0.05) were noted and retained in models with associations expressed as incidence rate ratios (IRR) with 95% confidence intervals. All analysis was performed using STATA v11.0 [*StataCorp* LP, College Station, Texas, USA].

## 3. Results

Demographic data for the 80 SLE participants and 41 controls (without autoimmune illness) are shown in Table 1 along with analysis of difference between groups (with *P* values). Groups were found to be well matched with no significant differences found in demographic characteristics. Participant mean age was 50 years for control group and 48 years with mean disease duration of 7.7 years (SD 6.2) for the SLE group. The majority of participants were Caucasian (97.5%) with 2 SLE participants and a single control participant identifying with Asian ethnicity. Both groups within the cohort reported educational levels to advanced or vocational level and above (62%), and an SES of either “above Australian average” or “Australian average” was reported in 110 (91%) of the combined cohort. Participants identifying as a current smoker did differ in percentage (control 2.4% : SLE 7.5%) however not significantly with Fisher's exact analysis. UV exposure through sunlight has impacts on vitamin D levels; therefore participants were asked whether they spent regular periods in the sun and also asked to estimate the average weekday and weekend hours spent in the outdoors (combined into an estimate of annual outdoor hours). Within our cohort, 68% of control participants as compared to 49% SLE participants reported that they experienced regular periods in the sun. This resulted in a calculated significant difference between the groups (*P* = 0.05). However this difference was not found when comparing the groups' calculated estimate of annual outdoor hours. The use of SS was reported in our cohort in similar proportions (control 78% : SLE 81%) reflecting general adherence to sun protective measures. Individual SS product sun protection factor (SPF) was not collected due to retrospective nature of study and multiple SS products used across the year; however health promotion advice within Australia recommends SS of a minimum SPF 30+ and the majority of SS products within Australia adhere to a minimum of SPF 30+ standard with broad spectrum UVA and UVB protection [25].

**Figure 2:**
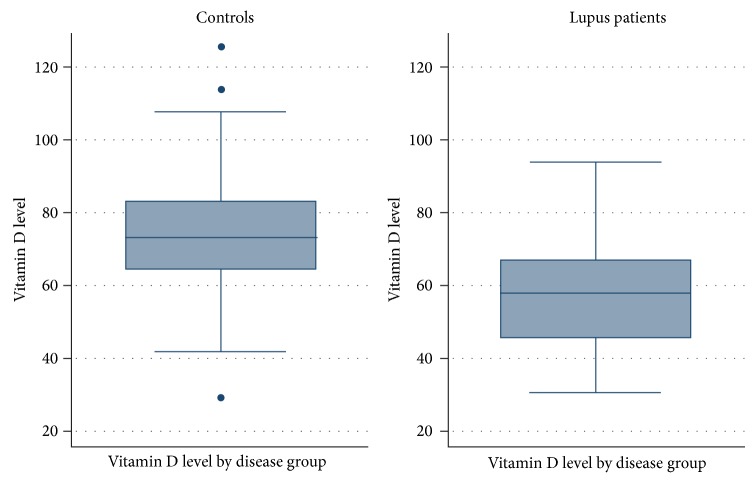
25(OH)D level comparison controls/SLE.

As highlighted in Figure 2, significant differences between groups were found for vitamin D 25(OH)D levels (mean: control 74 nmol/L (29.5 ng/mL); SLE 58 nmol/L (23.1 ng/mL), *P* = 0.04), 25(OH)D deficiency (*P* = 0.02), and levels categorised as abnormal (≤72.5 nmol/L) representing vitamin D levels considered to be both deficient and insufficient (*P* = 0.01). Deficiency level differences between groups were not observed when vitamin D supplementation use was considered; however, this did not extend to insufficient vitamin D 25(OH)D levels with insignificant differences found in unadjusted values but significant differences (*P* = 0.01) when participant use of supplementation was factored into analysis.

SLE participant individual ACR criteria were confirmed within audit phase of the study and included within association analysis to explore potential links between criteria and levels of 25(OH)D. No significant differences were found for any of the 11 ACR criteria in cross analysis with 25(OH)D levels or any subcategory of 25(OH)D levels. Due to reports of leucopoenia being a potential consequence of low vitamin D levels [3] a separate analysis via Fisher exact test was undertaken; however the association was not confirmed within our cohort.

The potential for pharmaceutical regimes to also be associated with altered 25(OH)D levels was tested with reference to quantified 25(OH)D levels and also 25(OH)D categories of deficient, insufficient, and a combined abnormal level. Medications and supplements were analysed as single variables and also as combined groups of variables as in ITM. It is of interest that hormone supplementation (*P* = 0.01) and combined ITM group (*P* = 0.05) and prednisolone (*P* = 0.03) were all associated with deficient levels of vitamin D. Association *P* values between 25(OH)D level, ACR criteria analysis, and medications of interest are outlined in Table 2.

Vitamin D deficiency in SLE has also been associated with higher prevalence with renal involvement [3, 26], photosensitive rash [26–28], leucopoenia [3], and arthritis [27, 28] as part of their diagnosis ACR symptom spectrum as well as flare symptoms. However within our cohort no significant association could be found with any ACR criteria and 25(OH)D levels.

ANA positivity, using a cutoff of ANA 1 : 80, was found in 80 (66%) of our cohort (control (17 : 41%) and SLE (63 : 79%)). Unsurprisingly, a significant difference (*P* = 0.00) indicates that participants with ANA positivity are more likely to be diagnosed with autoimmune SLE. Likewise, two-sample *t*-test analysis of ANA positivity and 25(OH)D levels clearly demonstrates that lower 25(OH)D levels are associated with a greater risk of autoimmunity (Table 3). Our study shows that higher levels of 25(OH)D offer a significant protective effect against the expression of SLE as an illness. Of the 34 participants showing a diagnostic deficiency of 25(OH)D, 79% (27) also had ANA positivity versus ANA positivity rate of 61% (53) in the absence of 25(OH)D deficiency. Our results highlight this association further with logistic regression models indicating that for every twofold rise in vitamin D level, the odds ratio for autoimmunity (ANA positivity) drops to 36% of the odds ratio (OR) that existed for the baseline level.

The mean number of SRF in the SLE participants was 6.8 with 12 participants reporting no flares over the study period. SRF-day counts were calculated from the estimated number of SRF that had occurred within the preceding 12 months to interview and the estimated length of each event. Total SRF-day counts ranged from 0 to 240 days (mean 29.2 ± 39). Two SLE participants had experienced major flares requiring extended periods in hospital, whilst 9 experienced SRF monthly and 2 on a weekly basis. The flare evaluation phase did not link individual SRF events with individual symptoms; therefore the count could represent an unresolved single symptom SRF and/or multiple events representing exacerbation of multiple or different symptoms. This study was retrospective and undertaken outside clinical assessment appointments, so validated clinic-based flare assessment tools were not able to be used.

The final pathway examined involved modelling SRF-day counts as the outcome variable and independent variables including ANA 1 : 80 and ACR criteria. Due to overdispersion of SRF-day counts, a negative binomial model was undertaken. The analysis was adjusted for demographic and social factors that could exhibit confounding effects for 25(OH)D levels (age, diagnosis years, BMI, smoking, stress, vitamin D and hormone supplements, outdoor hours, and use of UV barrier products (FMSS)). Those factors significant in univariate analysis were included in the final multivariate model. Statistically significant associations for both univariate and multivariate models are listed within Table 4.

No link was found between SRF-days and 25(OH)D levels or any sub categorisation of 25(OH)D levels indicating that whilst lower vitamin D levels may influence autoimmune diagnosis as defined by ANA-positivity, the lower levels do not influence flare risk or frequency. Protective associations were found for the use of UV barrier products of FMSS as indicated by OR and *P* values in both univariate and multivariate models. However, despite a significant association between ANA positivity (*P* = 0.007), the ACR criterion of arthritis (*P* = 0.003), and SRF frequency, this association was in a negative direction with OR of 0.48 and 0.51, respectively.

## 4. Discussion

### 4.1. Vitamin D Levels of Deficiency

Deficiency in vitamin D levels is often reported in general populations and is increasingly linked to increased incidence of autoimmune illnesses such as SLE and other musculoskeletal illnesses [12, 29]. General annual Australian population rates of deficiency are reported at 6% [30], with an estimate of 23% having levels below recommended “normal” levels (insufficient + deficient). Rates of deficiency vary significantly across seasons with rates in summer being lower than those in winter. Summer rates in the state of NSW are 19% with little variation with gender [30]. The proportion of 25(OH)D deficient but otherwise healthy individuals within our cohort was 14.6% as compared to our SLE group with a deficiency in 35.0%. Significant differences between our cohort groups were found and are reflective of similar findings internationally [26, 27]; however the proportion of deficiency found within our cohort differed from other studies with deficiency proportions within the SLE patients of 8.2% [31] to 20% [3, 14] although one study reported prevalence as high as 67% [32]. Variations in deficiency prevalence may be derived from population differences including geographic location, diet, social and economic factors, and population ethnicity. Significantly higher deficiency levels are often reported within individuals of African, Asian, and African American background and, in particular, persons with darker skin complexions [28, 33, 34].

Levels of insufficiency within our cohort were found in both healthy (46.3%) and SLE (48.8%) groups without adjustment for supplementation. This finding was considerably reduced when adjusted for supplement use. Insufficiency is not an unusual finding in patients with SLE even with supplementation [31, 35] with suggestions of a worldwide vitamin D insufficiency epidemic with levels falling 20% in 2002–2012 [36]. Our results suggest that within the Australian population there is also a high proportion of women with inadequate levels of 25(OH)D at either a deficient or insufficient level, with this inadequacy heightened within the autoimmune SLE population. Further to this the benefits of supplementation, which did not significantly differ across the groups, appear to be reduced within the autoimmune SLE population, supporting the need for additional or nonstandard measures of support and monitoring. Beneficial supplementation to raise 25(OH)D levels to above insufficient levels within an SLE patient has been reported within a phase 1 trial to require daily doses of 2000 IU as opposed to current standard doses of 600–800 IU [37].

### 4.2. Expression of Autoimmunity (ANA 1 : 80 Positivity)

It is reported that 13–45% of healthy individuals may have ANA positivity during their lifetime [38–40]. Prevalence is reported to be higher with aging and in women. High variation in frequency ranges has also been found across various geographical groups and with methods [41]. Within our cohort, ANA positivity with a dilution cutoff at 1 : 80 was found in 41% of our controls (without autoimmune illness) as compared to 79% in ACR confirmed SLE patients with a significance difference in prevalence found between the two groups. Prevalence within healthy controls was at the upper end of our expectation and reflected the high proportion of older individuals (9/17 = 53% over 50 years) in the study. A high ANA positivity prevalence had previously been found in healthy controls in a Hunter, NSW community cohort study Boyle [42].

### 4.3. Vitamin D and Expression of Autoimmunity (ANA 1 : 80 Positivity)

The log2 logistic model related 25(OH)D levels to probability of ANA positivity. Substitution allowed absolute odds values to be calculated. For a 25(OH)D level of 40 nmol/L, the probability of ANA positivity is 79% (odds 3.67 : 1), whilst a 25(OH)D level of 80 nmol/L is associated with an ANA positivity probability of only 57% (odds 1.32 : 1), equating to 36% of the odds of ANA positivity for the lower vitamin D level of 40 (equivalent to a relative risk reduction of 67%). This confirms that as vitamin D levels increase, probability of autoimmune disease expression decreases. This finding, with particular reference to vitamin D deficiency and expression of an autoimmune disease (ANA 1 : 80), is in line with established literature [43, 44].

### 4.4. Clinical Autoimmunity (ANA 1 : 80 Positivity) and Self-Reported Flare

The negative association between serological magnitude of autoimmunity and SRF frequency was unexpected but consistently demonstrated throughout data subanalysis of ANA patterns (homogeneous only), titres, and a restricted analysis of ANA-positive participants with concurrent ENA and/or DNA positivity. ANA patterns were of particular interest due to reports of dense fine speckled 70 (DFS70) antigen staining, as a potential marker of protection against autoimmunity [39, 45, 46].

This concords with evidence for SLE and autoimmunity representing a multistep process proceeding from self-tolerance to serological “benign” autoimmunity (step 1), followed by later conversion of serological “silent” autoimmunity, to overt autoimmune disease (SLE) (step 2) [47]. Current evidence [40] supports the contention that the triggers invoking each of these transitions (steps 1 and 2) are distinct for each step, with basic immunomodulating factors such as vitamin D levels being associated with transition across the first step in autoimmune pathogenesis and with other factors, perhaps impacting upon maintenance of peripheral immune regulation (e.g., hormones, sunlight) being important in driving step 2.

Given the virtually universal requirement for ANA positivity to exist before a clinical diagnosis of SLE is made, there is an inherent incorporation bias operating in any study attempting to tease out relative pathogenic contributions of various determinants to either “benign” (serological) or “overt” (clinical) autoimmunity: this is because the presence of ANA positivity increases the likelihood of a SLE diagnosis being made. This phenomenon occurs despite the decline in immune function with aging (immunosenescence), which is often associated with a rise in ANA expression and titres, in the absence of any clinical autoimmune disease presence.

Given the results of separate studies defining links between environmental chemical exposure and SRF frequency [48, 49], the proposal that different triggers operate for each transitional step seems appropriate and consistent with available data.

The phenomenon of “protective” autoimmunity is well established, with antinuclear antibodies having been shown to operate to limit immune-related pathology in a range of settings. Examples of protective autoimmunity include (i) expression of ENA along with ANA protecting against severe renal lupus [50]; (ii) antibodies to high-mobility group protein B1 (HMGB1) ANA, attenuating disease in mouse models and providing survival benefit in patients with septic shock [40], suggesting that infection-induced antibodies may be beneficial in some instances; and (iii) “DFS- (dense fine speckled-) 70” antibodies (previously labelled as a type of homogeneous ANA with speckling) which are increasingly reported in clinical studies, and there is a reported reduction in prevalence of lupus-related autoimmune syndromes in this patient subgroup [38, 39].

Our study's findings are consistent with a proposed role of ANA in offering protection against lupus flares, and, indeed, expression of some ANAs may be a marker of adaptive regulatory immune function as an allostatic mechanism.

### 4.5. Vitamin D and Self-Reported Flare

Despite the clear autoimmune predisposition conferred by low 25(OH)D levels, we could find no significant association between 25(OH)D levels and frequency of SRF in SLE participants. A lack of correlation has also been reported in other vitamin D/activity studies [31, 51]. In contrast, a large number of studies have found inverse relationships between low levels of 25(OH)D and SLE activity measures [23, 26, 34, 45]. Within our study we propose that a number of factors may explain our lack of association, including (i) lack of statistical study power; (ii) retrospective nature of SRF; and (iii) reasoning that vitamin D deficiency may predispose to disease expression without influencing its severity or behaviour once established. As discussed above, the two-step model of conversion from tolerance to “benign” autoimmunity and then to “clinical” autoimmunity is consistent with separate roles for different determining factors in the expression of aberration. Thus, whilst we have shown that vitamin D levels have impact upon step 1 (the expression of “benign” autoimmunity), we must posit other factors that prompt the second step of autoimmune disease development, possibly triggered by other environmental or hormonal factors [48, 49, 52].

## 5. Study Strengths and Weaknesses

This study provides retrospective data within the context of a relatively homogenous Caucasian population with a validated SLE diagnosis according to ACR criteria. The retrospective design would limit firm establishment of casual relationships. The study was undertaken outside routine clinical management appointments with data collection from a nonclinician; therefore SLE flare data collected was from a self-reported patient perspective rather than according to standard disease activity measures. To minimise bias, the methods followed a standardised protocol which included a flare definition [21] and structured interview method [18]. Individual SRF events were not matched to specified symptoms and without clarification of whether full resolution of flare occurred each time an event was reported; however only a few participants reported flare frequency rates greater than monthly. The self-reported nature of flare assessment may have resulted in an overestimation of actual days and events recorded creating potential bias against detection of a significant association with vitamin D levels.

Despite collection of samples within a single spring season, the dynamic and fluctuating nature of vitamin D levels [34] coupled with the retrospective assessment of SRF may have resulted in missed associations between 25(OH)D and flare frequency.

The capacity for extrapolation of results from this study is limited by the lack of diversity in ethnicity and gender within our study population. The results should be viewed as of a pilot nature with the need for a comprehensive prospective study protocol documenting vitamin D levels over time along with assessment of disease activity and occurrence of flares potentially adding to evidence of any associations.

## 6. Conclusion

In summary, our paper reinforces existing literature supporting a link between vitamin D deficiency and the expression of autoimmune phenomena (ANA positivity) and clinical conditions (such as SLE). In contrast, there was no demonstrable association between vitamin D levels and flare frequency, consistent with the proposed independent influence of other flare-inducing factors. The results indicate that SLE patients are at a high risk of vitamin D deficiency and vitamin D supplementation along with regular monitoring should be a consideration as part of individual patient health management plans.

## Figures and Tables

**Table 1:** Characteristics of participant groups.

	Control (*N* = 41)	SLE ACR 4+ (*N* = 80)	Difference between groups

	Mean ± SD	Mean ± SD	*P* value

Age (years)	49.8 ± 12.4	47.7 ± 13.5	1.0
Diagnosis (years)		7.7 ± 6.2	
Outdoor hours per year	718.2 ± 506	490.5 ± 433	0.99
Body mass index score	25.9 ± 4.8	27.4 ± 5.6	1.0
Makeup (FMSS) days per year	307 ± 227	291 ± 214	0.99
Vitamin D 25(OH)D ng/mL	29.5 ± 8.2	23.1 ± 6.3	**0.04**
Vitamin D 25(OH)D nmol/L	73.7 ± 20.6	57.8 ± 15.7	**0.04**

	no. (%)	no. (%)	*P* value

Educational background			0.43
Socioeconomic status			0.48
Above average	10 (24.4)	15 (18.8)	
Average	29 (70.7)	56 (70.0)	
Below average	2 (4.9)	9 (11.3)	
Current smoker	1 (2.4)	6 (7.5)	0.42
Regular sun	28 (68.3)	39 (48.8)	**0.05**
Use sunscreen	32 (78)	65 (81.3)	0.81
Use immunotherapy medications		67 (83.8)	
Use of vitamin D supplements	11 (26.8)	29 (36.3) 0	0.32
Use of hormone supplements	10 (24.4)	30 (37.5)	0.16
Deficient 25(OH)D	6 (14.6)	28 (35.0)	**0.02**
Insufficient 25(OH)D	19 (46.3)	39 (48.8)	0.85
Abnormal 25(OH)D	25 (61)	67 (83.8)	**0.01**
Deficient 25(OH)D with supplements	2 (4.9)	13 (16.3)	0.09
Insufficient 25(OH)D with supplements	1 (2.4)	15 (18.8)	**0.01**
Abnormal 25(OH)D with supplements	3 (7.3)	23 (28.8)	**0.01**
ANA ≥1 : 80 ratio	17 (41.5)	63 (78.8)	**0.00**

Vitamin D categories: deficiency (50 nmol/L (≤20 ng/mL)), insufficiency (52.5–72.5 nmol/L (>20.0 ≤ 29 ng/mL)), and abnormal (≤72.5 nmol/L (≤29 ng/mL)) [3].

**Table 2:** SLE participant differences: ACR, medications, and 25(OH)D status.

*N* = 80	Number (%)	*P* value	Number (*P* value)	Number (*P* value)
		Vit. D level	Vit. D deficient	Vit. D insufficient
Clinical ACR SLE features				
Malar rash	57 (71.3)	0.54	16 (0.07)	31 (0.14)
Discoid rash	3 (3.8)	0.23	2 (0.28)	1 (1.0)
Photosensitivity	43 (53.8)	0.52	11 (0.07)	23 (0.38)
Oral/nasal ulcers	29 (36.3)	0.12	10 (1.0)	12 (0.36)
Arthritis	63 (78.8)	0.63	21 (0.58)	31 (1.0)
Serositis	20 (25.0)	0.91	6 (0.79)	10 (1.0)
Renal disorder	37 (46.3)	0.49	16 (0.17)	15 (0.19)
Neurological disorder	33 (41.3)	0.67	12 (1.0)	17 (0.82)
Haematological disorder	39 (48.8)	0.95	14 (1.0)	20 (0.82)
Leucopoenia	18 (22.5)	0.57	7 (0.78)	10 (0.6)
Immunologic disorder	27 (33.8)	0.42	11 (0.47)	14 (0.81)
Antinuclear antibody ≥1 : 80	63 (78.8)	0.27	24 (0.39)	28 (0.18)
Medications				
Hormones	30 (37.5)	0.23	**5 (0.01)**	16 (0.65)
ITM	67 (83.8)	0.56	**20 (0.05)**	36 (0.07)
Prednisolone	34 (42.5)	0.31	**7 (0.03)**	21 (0.07)
Vitamin D + prednisolone	15 (18.8)	0.71	4 (0.56)	9 (0.4)

Vitamin D categories: deficiency (50 nmol/L (≤20 ng/mL)) and insufficiency (52.5–72.5 nmol/L (>20.0 ≤ 29 ng/mL)) [3].

**Table 3:** Tests of association ANA80 and log⁡⁡(2) transformed 25(OH)D levels.

Test	Group (*n*)	OR	*P* > |*Z*|	Mean (SD)	95% CI	
Two-sampled *t*-test 25(OH)D by ANA80	Control, SLE (121)		**0.014**			
	ANA −ve (41)			68.9 (21.5)	62.1	75.7
	ANA +ve (80)			60.3 (16.9)	56.5	64.0

Two-sampled *t*-test log⁡⁡(2)25(OH)D by ANA80	Control, SLE (121)		**0.017**			
	ANA −ve (41)			6.0 (0.45)	5.9	6.2
	ANA +ve (80)			5.9 (0.42)	5.8	5.9

Two-sampled *t*-test log⁡⁡(2)25(OH)D by group	Cohort (121)		**0.00**			
	Control (41)			6.1 (0.42)	6.0	6.3
	SLE (80)			5.8 (0.40)	5.7	5.9

Linear regression log⁡⁡(2)25(OH)D ANA80	Control, SLE (121)		**0.029**		−0.35	−0.02
	ANA −ve (41)					
	ANA +ve (80)					

Logistic regression ANA80 log⁡⁡(2)25(OH)D	Control, SLE (121)	0.36	**0.032**		0.14	0.92
	ANA −ve (41)					
	ANA +ve (80)					

**Table 4:** Negative binomial regression for self-reported flare days (SRF-days) and independent variables.

	*n *	Mean (SD)	95% CI	
SRF number (year)	80	6.8 (9.7)	4.6	8.9
SRF-days (year)	80	29.2 (39)	20.5	37.9

Univariate model	OR	*P* > |*Z*|	95% CI	

25(OH)D	0.99	0.27	0.97	1.0
Deficient 25(OH)D	1.33	0.37	0.72	2.46
Insufficient 25(OH)D	0.93	0.82	0.52	1.67
Abnormal 25(OH)D	1.53	0.25	0.75	3.11
Makeup (FMSS)	1.0	**0.002**	1.0	1.0
Diagnosis years	0.97	**0.024**	0.94	1.0
ANA 1 : 80	0.42	**0.008**	0.22	0.80
dsDNA	0.29	**0.009**	0.12	0.74
ENA	0.54	**0.027**	0.31	0.93
Arthritis	0.48	**0.022**	0.26	0.90
Immunological disorder	0.49	**0.009**	0.29	0.84

Multivariate model	OR	*P* > |*Z*|	95% CI	

Makeup (FMSS)	0.99	0.001	0.99	0.99
ANA 1 : 80	0.48	0.007	0.28	0.85
Arthritis	0.51	0.003	0.33	0.79
